# Involvement of AoMdr1 in the Regulation of the Fluconazole Resistance, Mycelial Fusion, Conidiation, and Trap Formation of *Arthrobotrys oligospora*

**DOI:** 10.3390/microorganisms11061612

**Published:** 2023-06-19

**Authors:** Yankun Liu, Xuewei Yang, Meichen Zhu, Na Bai, Wenjie Wang, Jinkui Yang

**Affiliations:** State Key Laboratory for Conservation and Utilization of Bio-Resources in Yunnan, Key Laboratory for Southwest Microbial Diversity of the Ministry of Education, School of Life Science, Yunnan University, Kunming 650032, Chinabaina@mail.ynu.edu.cn (N.B.);

**Keywords:** *Arthrobotrys oligospora*, multidrug resistance protein, fluconazole resistance, conidiation, trap formation

## Abstract

Multidrug resistance (Mdr) proteins are critical proteins for maintenance of drug resistance in fungi. Mdr1 has been extensively studied in *Candida albicans*; its role in other fungi is largely unknown. In this study, we identified a homologous protein of Mdr (AoMdr1) in the nematode-trapping (NT) fungus *Arthrobotrys oligospora*. It was found that the deletion of *Aomdr1* resulted in a significant reduction in the number of hyphal septa and nuclei as well as increased sensitivity to fluconazole and resistance to hyperosmotic stress and SDS. The deletion of *Aomdr1* also led to a remarkable increase in the numbers of traps and mycelial loops in the traps. Notably, AoMdr1 was able to regulate mycelial fusion under low-nutrient conditions, but not under nutrient-rich conditions. AoMdr1 was also involved in secondary metabolism, and its deletion caused an increase in arthrobotrisins (specific compounds produced by NT fungi). These results suggest that AoMdr1 plays a crucial role in the fluconazole resistance, mycelial fusion, conidiation, trap formation, and secondary metabolism of *A. oligospora*. Our study contributes to the understanding of the critical role of Mdr proteins in mycelial growth and the development of NT fungi.

## 1. Introduction

Currently, antibiotic resistance is a global problem in modern medicine. The cornerstone of bacterial defense against antibiotics is the multidrug resistance pump, which involves antibiotic resistance, toxin export, biofilms, and persistent cell formation [1]. Multidrug resistance (Mdr) proteins are ATP-binding cassette (ABC) transporters that play key roles in mediating fungal resistance to pathogenesis-associated stress [2]. Mdr1 is a membrane protein (efflux pump) responsible for the efflux of toxic substances that resist the external environment [3]. The transcriptional activation of the drug-efflux-encoding gene *mdr1* is a common pathway for the acquisition of fluconazole resistance in *Candida albicans* [4]. Overexpression of the *mdr1* gene has been shown to be responsible for fluconazole and panozole resistance in *Candida tropicalis* clinical isolates [5]. Similarly, *mdr1* expression was involved in the acquisition of azole resistance by *Aspergillus flavus* and *Aspergillus fumigatus* [6,7]. In contrast, the absence of *mdr1* in *Cryptococcus gattii* did not affect fluconazole resistance or virulence [8]. However, little is known about the roles of Mdr proteins in other fungi.

A predatory lifestyle is usually associated with animals such as eagles and wolves. However, researchers have found that predatory behavior also occurs in microorganisms [9]. Among them, nematode-trapping (NT) fungi are a group of fungi that are widely distributed in multiple environments and play essential roles in regulating nematode populations in soil [10]. NT fungi are capable of forming various trapping devices (traps) for nematode predation, including constricting rings and adhesive traps (adhesive nets, adhesive columns, and non-constricting rings) [11]. Trap formation for nematode predation allows a parasitic lifestyle; therefore, it also signifies a lifestyle transition [9,10,11,12]. The interactions between NT fungi and nematodes reflect a highly complex co-evolutionary relationship that has been in existence for hundreds of millions of years [13]. For example, the capacity of specific urea-releasing bacteria to induce the NT fungus *Arthrobotrys oligospora* to produce traps for nematode predation has been shown to lead to a change in lifestyle [14]. *A. oligospora* is a typical species used to investigate nematode–fungal interactions. Under nematodes and other stimulations, *A. oligospora* is able to form adhesive networks to feed on nematodes [15]. In recent years, studies of the growth, development, and differentiation of *A. oligospora* have gradually increased, demonstrating multiple signaling proteins and cellular processes involved in the vegetative growth, conidiation, and trap formation of NT fungi, such as autophagy [16,17,18], peroxisomes [19,20], G-protein and related signaling [21,22], and the AMPK [23] and MAPK pathways [24,25,26]. However, the function of Mdr proteins in NT fungi remains largely unknown. 

In this study, we identified an orthologous Mdr protein (AoMdr1) in *A. oligospora*. Its gene was disrupted using a homologous recombination technique, and its functions in fluconazole resistance, stress response, conidiation, and trap formation were characterized using phenotypic analysis and non-targeted metabolomics.

## 2. Materials and Methods

### 2.1. Strains, Plasmids, and Growth Conditions

A wild-type (WT) strain of *A. oligospora* (ATCC24927) and mutant strains were maintained in a potato dextrose agar (PDA) medium at 28 °C. The pCSN44 plasmid was used for the amplification of the hygromycin resistance gene (*hph*) preserved in *Escherichia coli* strain DH5a (Takara, Dalian, China); the pRS426 plasmid was used for the construction of the knockout vector [27]. The FY834 strain of *Saccharomyces cerevisiae* was used to construct the homologous recombinant vector for the knockout of the *Aomdr1* gene, which was cultured in yeast extract–potato dextrose (YPD) (10 g/L yeast extract, 20 g/L peptone, and 20 g/L dextrose) [28]. The FY834 strain with the correct vector was selected using an SC-Ura medium (2 g/L synthetic drop-out mix minus uracil without a yeast nitrogen base, 26.7 g/L drop-out base with glucose, and 20 g/L agar). In the regeneration of protoplasts, a PDAS medium (PDA supplement with 0.6 M of sucrose) was used to select the putative transformants [23]. *Caenorhabditis elegans* was incubated in an oatmeal–water medium at room temperature for the bioassay. *A. oligospora*, *S. cerevisiae*, and *C. elegans* were obtained from the Microbial Library of the Germplasm Bank of Wild Species from Southwest China. 

### 2.2. Sequence and Cluster Analysis of AoMdr1

The *A. oligospora* Mdr1 protein AoMdr1 (AOL_s00215g705) was identified based on the orthologs in the model fungi *Aspergillus nidulans* (Q9Y8G1.1) and *A. fumigatus* (KAF4260332.1). The isoelectric point and molecular weight of AoMdr1 were analyzed using the pI/Mw online tool (http://www.expasy.ch/tools/pi_tool.html) (accessed 20 March 2023). The conserved domains were predicted using the Interproscan website. We used DNAman software (version 6; Lynnon Biosoft, San Ramon, CA, USA) for a comparison of the sequence similarities of the Mdr1 orthologs from different fungi [29]. A phylogenetic tree was constructed using the neighbor-joining (NJ) method using MEGA software (version 7) [30].

### 2.3. Deletion and Verification of Aomdr1

In this study, we disrupted *Aomdr1* using a homologous recombination technique and the *hph* resistance gene. We replaced the *Aomdr1* gene, as has been previously described [31]. The primer pair of AoMdr1-5F/5R and AoMdr1-3F/3R (Table S1) was used for the amplification of the upstream and downstream homologous arms; primer Hph5F/3R was used for the amplification of the *hph* gene, using pSCN44 as the template. The resulting three PCR fragments and the *Eco*RI/*Xho*I linearized vector pRS426 were transformed into FY834 strains using the PEG/CaCl_2_-mediated transformation method. These were inoculated in the SC-Ura medium for the selection of the recombinational strains. Finally, the recombinant vector was selected to amplify the full-length disruption fragment using AoMdr1-5F/3R (Table S1). The full-length fragment was transformed into protoplasts of *A. oligospora* and selected using a PDAS medium containing 200 μg mL^−1^ hygromycin B, as has been previously described [31]. A double validation of the transformants using PCR amplification and quantitative reverse transcription PCR (RT-qPCR) was performed to obtain positive transformants. Primers AoMdr1-PF and AoMdr1-PR (Table S1) were designed and used for the PCR amplification. In the RT-qPCR analysis, the total RNAs of the WT and the positive transformants were extracted using a Trizol reagent (Invitrogen, Carlsbad, CA, USA), and a PrimeScript RT reagent kit (Takara, Shiga, Japan) was used for the reverse transcription. The relative level of transcription for each gene was calculated using the threshold cycling method (2^−∆∆CT^), with the β-tubulin gene as an internal reference [32,33].

### 2.4. Comparison of Mycelial Growth and Cell Nuclei

Both the WT and mutant strains were inoculated in three media—PDA, TG, and TYGA—and the colony diameters were recorded for a comparison of the mycelial growth rates at 24 h intervals [34]. The mycelia were collected from the PDA culture for 5 days at 28 °C; 20 μg/mL calcofluor white (CFW, Sigma-Aldrich, St. Louis, MO, USA) was used for the staining to observe the mycelial morphology and septa. The mycelia were stained with 20 μg/mL 4’,6-diamidino-2-phenylindole (DAPI, Sigma, St. Louis, MO, USA) for 15 min and observed using inverted fluorescence microscopy (Carl Zeiss, Oberkochen, Germany) [35]; 50 photos were randomly taken for cell nucleus counting.

### 2.5. Observation of Mycelial Fusion and Lipid Droplet (LD) Accumulation

To examine the effect of *Aomdr1* on the mycelial fusion phenomenon, the WT and ∆*Aomdr1* mutants were inoculated in a nutrient-deficient water agar (WA) medium (20 g/L agar), a minimal medium (MM) (0.01 g/L FeSO_4_·7 H_2_O, 2 g/L NaNO_3_, 20 g/L glucose, and 20 g/L agar), a nutrient-rich PDA medium, and WA + N (WA supplemented with 300 nematodes) for 5 days. The mycelia were then stained with 20 μg/mL CFW [33]. The mycelia were also stained with 10 μg/mL boron dipyrromethene dye (BODIPY, Sigma-Aldrich); after 5 days of PDA incubation, the mycelial sample was then observed using inverted fluorescence microscopy.

### 2.6. Stress Response Assays

The stress responses of the WT and ∆*Aomdr1* mutants to chemical stressors were assessed in a TG medium supplemented with a cell-wall-perturbing agent (sodium dodecyl sulfate (SDS)) and osmotic agents (NaCl and sorbitol) for 5 days at 28 °C [36]. To test the fungal response to heat shock, the fungal strains were incubated on a TYGA plate for 2 days, then transferred into 28, 32, 36, and 40 °C conditions, respectively, for 6 h post-incubation, then moved to 28 °C conditions to continue incubation for a total of 5 days [37]. To investigate the tolerance of AoMdr1 to fluconazole (Flc), the PDA medium was used as a control and different concentrations of Flc solution (10, 20, and 30 μg/mL) were added to the PDA for the growth observations of the WT and mutant strains. The relative growth inhibition (RGI) values of the fungal strains were calculated, as has been previously described [38]. The assays for each strain were repeated three times.

### 2.7. Comparison of Conidial Production, Trap Induction, and Bioassay

To compare the sporulation abilities of the WT and ∆*Aomdr1* mutants, all strains were cultured in a corn meal–yeast extract (CMY) medium at 28 °C for 14 days. The spores were eluted with 20 mL of sterile distilled water, filtered, and counted using a microscope [39]. The spores were stained with 20 μg/mL CFW, and the spore morphology was observed using inverted fluorescence microscopy [40]; 30 photos were randomly obtained for the morphological statistics.

To induce trap production, approximately 2 × 10^6^ conidia of the WT and mutant strains were added to 6 cm WA plates for germination. After 3 days of growth, approximately 300 of *C. elegans* were added to each plate to induce trap production [25]. After the addition of the nematodes, the nematode mortality and the number of traps in the plates were counted at 12 h intervals. Three replications of each strain were performed. To detect protease activity, the WT and ∆*Aomdr1* mutants were inoculated in potato dextrose (PD) broth. The fermentation broth was collected after incubation at 28 °C and 180 rpm for 7 days; casein plates were used for the protease activity assay [41].

### 2.8. Scanning Electron Microscopy (SEM) and Transmission Electron Microscopy (TEM) Assays

The WT and mutant strains were inoculated on CMY plates (for mycelial and spore observation) and grown for 5 days. The mycelia were collected for SEM observation [42]. The LDs in the mycelia were observed using TEM for all strains, which were incubated on PDA plates for 5 days [15]. To compare the trap morphology, 2 × 10^6^ conidia of WT and mutant strains were incubated on WA plates for 3 days and 300 nematodes were added to induce growth over 48 h. The mycelia and traps were then collected for SEM observation [42].

### 2.9. Metabolomics Profile Analysis

PD broth fermentation was obtained from the WT and ∆*Aomdr1* mutants after incubation for 7 days. The hyphal dry weight was recorded, and equal volumes of ethyl acetate were added, mixed, and then ultrasonicated twice (20 min/time). The liquid-phase layer of the ethyl acetate was spun using a spinner, and chromatographic-grade methanol was used to dissolve the compounds, which were filtered through a 0.22 μm membrane filter and then analyzed with LC-MS [43]. Compound Discoverer 3.0 software (Thermo Fisher Scientific, Miami, FL, USA) was used for the analyses of different compounds in all strains. Thermo Xcalibur software (version 3.0, Thermo Fisher Scientific) was used for a comparison of the total metabolic profiles of all strains [44].

### 2.10. Statistical Analysis

All experimental data were obtained by repeating the measurements three times, and the data were statistically analyzed (one-way ANOVA) using Prism 8.0 (GraphPad Software, San Diego, CA, USA). Data were considered to be significantly different if *p* < 0.05.

## 3. Results

### 3.1. Sequence and Phylogenetic Analyses of AoMdr1

Aomdr1 encoded a polypeptide, containing 1343 amino acid residues, with an isoelectric point of 6.26 and a molecular mass of 143.84 kDa. A phylogenetic tree was constructed based on the amino acid sequences of Mdr1 homologs from ten fungal species, including *A. oligospora*, using the NJ method. These Mdr1 homologs were divided into two clades, and AoMdr1 and orthologs from five other NT fungi were clustered into a single clade (Figure S1A). Mdr1 homologs from different filamentous fungi shared two ABC_tran and two ABC_membrane structural domains. AoMdr1 had a high degree of similarity with orthologs from the four NT fungi *Arthrobotrys flagrans* (95.9%), *Dactylellina haptotyla* (87.9%), *Arthrobotrys entomopaga* (84.4%), and *Drechslerella brochopaga* (84.6%). It had moderate similarity (57.4% to 60.1%) with the orthologs of other filamentous fungi such as *A. nidulans* (58%) and *A. fumigatus* (57.4%) (Figure S1A).

### 3.2. AoMdr1 Plays a Vital Role in Hyphal Septa and the Number of Cell Nuclei

**Figure 1:**
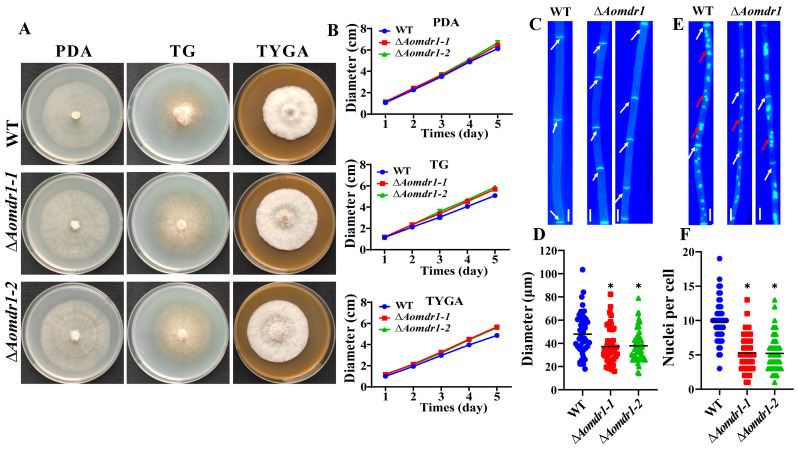
Comparisons of mycelial growth, septa, and nuclei of the WT and ∆*Aomdr1* mutants. (**A**) Colony morphologies of fungal strains cultured at 28 °C for 5 days. (**B**) Comparisons of mycelial growth rates. (**C**) Observations of mycelial septa on PDA plates. White arrows indicate mycelial septa; scale bar: 2 μm. (**D**) Comparison of mycelial cell lengths. (**E**) Mycelial cell nuclei on PDA plates; scale bar: 2 μm. White arrows indicate mycelial septa and red arrows indicate nuclei. (**F**) Comparison of the number of nuclei. Asterisks indicate significant differences between the ∆*Aomdr1* mutant and WT strains (Tukey HSD, * *p* < 0.05).

Two positive transformants were obtained using the homologous recombination method, as shown in Figure S1B. These were verified with PCR and RT-qPCR methods (Figure S1C,D). The colonies of ∆*Aomdr1* mutants were slightly larger than those of the WT in all three media (PDA, TG, and TYGA), especially in the TYGA medium (Figure 1A,B). The mycelial septa of the mutants were considerably greater than those of the WT strain (Figure 1C,D). The mycelial cells of the WT strain contained 3–16 nuclei (9.86 on average), whereas those of the mutant strain contained 1–13 nuclei (5.24 on average) (Figure 1E,F).

### 3.3. AoMdr1 Regulates LD Accumulation and Hyphal Fusion under Nutrient-Deprived Conditions

**Figure 2:**
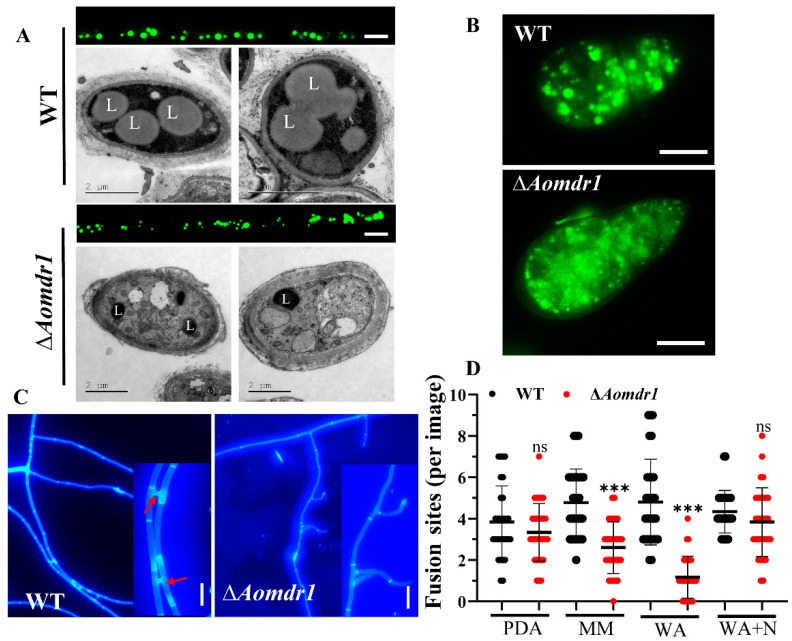
Comparisons of LD accumulation and mycelial fusion in WT and ∆*Aomdr1* mutant strains. (**A**) LD accumulation in mycelial cells was observed with BODIPY staining (upper pane) and TEM images (lower pane). L indicates LDs; scale bar: 5 µm. (**B**) BODIPY staining of LDs in conidia; scale bar: 10 µm. (**C**) Observation of hyphal fusion in WA plates. The red arrows indicate the hyphal fusion sites; scale bar: 5 µm. (**D**) Comparison of the number of hyphal fusion sites under different media. The WT and mutant strains were observed using CFW staining for hyphal fusion after 5 days of incubation in PDA, WA, MM, and WA + N media, and 30 random photographs were used to count the number of hyphal fusion sites. The representative images in (**A**–**C**) were chosen from ∆*Aomdr1-1* and ∆*Aomdr1-2* mutants. Asterisks indicate significant differences between the ∆*Aomdr1* mutant and WT strains (Tukey HSD; *** *p* < 0.001).

After staining with BODIPY dye, the volume of the LDs in the mutants became smaller than in the WT strain (Figure 2A,B). The TEM images also proved that there was a greater LD accumulation in the WT strain than in the ∆*Aomdr1* mutants (Figure 2A). The CFW staining results show that the deletion of the *Aomdr1* gene resulted in impaired mycelial fusion, which was related to the different media. On the nutrient-deficient WA and MM media plates, the mycelial fusion of the ∆*Aomdr1* mutants was significantly lower than that of the WT strain; however, in the nutrient-rich PDA and WA + N plates, the mycelial fusion of the ∆*Aomdr1* mutants was not obviously different from that of the WT strain (Figure 2C,D).

### 3.4. AoMdr1 Afects the Stress Responses of Fluconazole and Chemical Reagents

**Figure 3:**
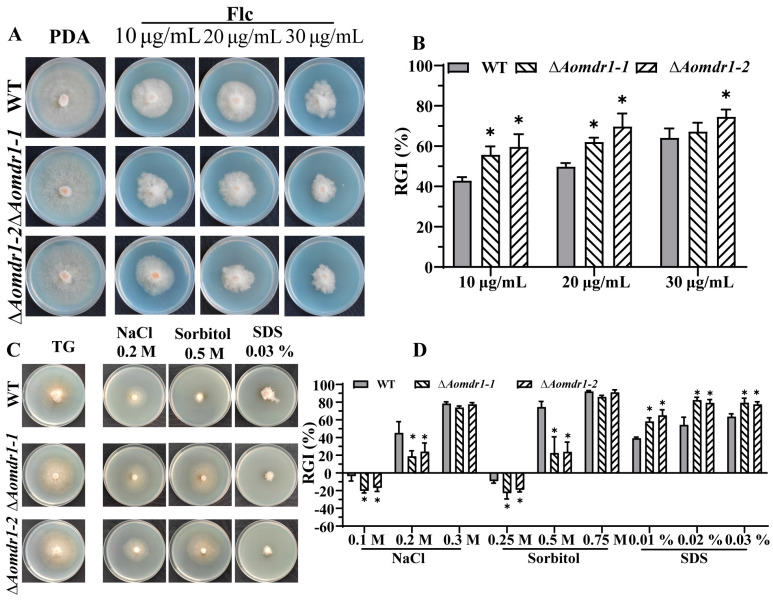
Comparisons of stress responses for fluconazole resistance, osmotic pressure, and SDS treatment. (**A**) Growth of WT and mutant strains under different concentrations of fluconazole. (**B**) RGI values of WT and mutant strains under fluconazole treatment. (**C**) Comparisons of mycelial growth after treatment with 0.2 M of NaCl, 0.5 M of sorbitol, and 0.03% SDS. (**D**) RGI values of WT and mutant strains under chemical stresses. Asterisks indicate significant differences between the ∆*Aomdr1* mutant and WT strains (Tukey HSD; * *p* < 0.05).

A comparison of the resistance to fluconazole showed that the ∆*Aomdr1* mutants were more sensitive than the WT strain. Specifically, the RGI values of the ∆*Aomdr1* and WT strains were 59.6% and 42.8%, respectively, under the Flc treatment at a concentration of 10 μg/mL and 69.7% and 49.8%, respectively, under the Flc treatment at a concentration of 20 μg/mL (Figure 3A,B). Under NaCl and sorbitol treatments, the ∆*Aomdr1* mutants showed reduced RGI values compared to the WT strain. The RGI values of the ∆*Aomdr1* and WT strains were 21.4% and 45.2%, respectively, under the 0.2 M NaCl treatment and 23.1% and 75.5%, respectively, under the 0.5 M sorbitol treatment (Figure 3C,D). The mutant strains showed higher RGI values in the ∆*Aomdr1* mutants than in the WT strain under SDS stress; for example, the RGI values of the ∆*Aomdr1* and WT strains were 80.7% and 54.4%, respectively, under the 0.02% SDS treatment (Figure 3C,D). The TEM picture showed that the mycelial cells of several mutant strains demonstrated plasma-wall separation (Figure S2A). The mutant strains became more sensitive under a stress of 40 °C, whereas there was no significant difference at 32 or 36 °C (Figure S2B).

### 3.5. AoMdr1 Impairs Sporulation and Spore Morphology

**Figure 4:**
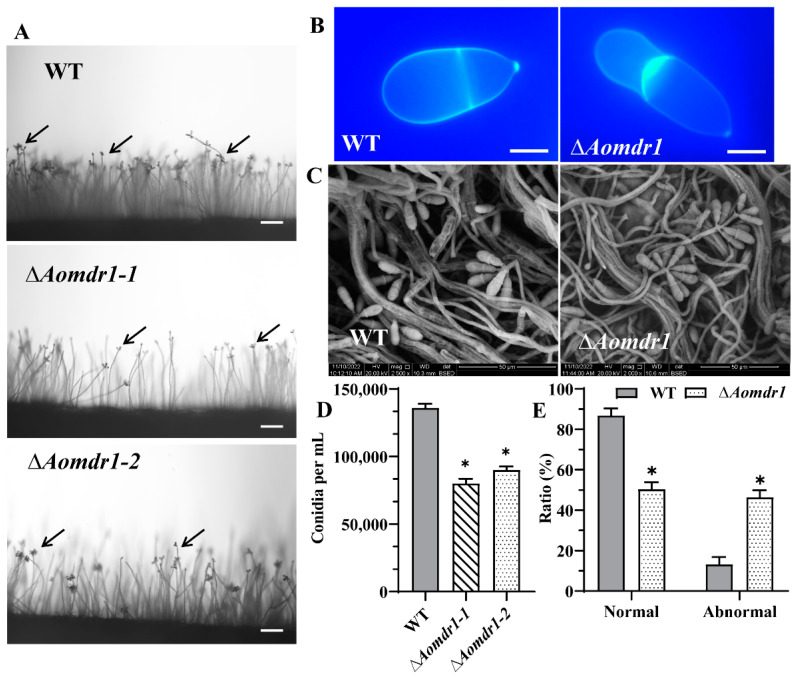
Comparisons of sporulation and spore morphology. (**A**) Observation of conidiophores of ∆*Aomdr1* mutant and WT strains; scale bar: 50 µm. (**B**) CFW staining of spores of the ∆*Aomdr1* mutant and WT strains; scale bar: 10 µm. The black arrows indicate the conidia. (**C**) SEM observations of spore morphologies of ∆*Aomdr1* mutant and WT strains. The representative images in (**B**,**C**) were chosen from ∆*Aomdr1-1* and ∆*Aomdr1-2* mutants. (**D**) Comparison of the spore yield between the mutant and WT strains. (**E**) Percentages of normal and abnormal spores. A total of 100 random spores were used to calculate the ratio of normal and abnormal spores. Asterisks indicate significant differences between the ∆*Aomdr1* mutant and WT strains (Tukey HSD; * *p* < 0.05).

The deletion of *Aomdr1* did not alter the production of conidiophores in the Δ*Aomdr1* mutants (Figure 4A), whereas the spore morphology was deformed (Figure 4B,C). Of the Δ*Aomdr1* mutant spores, 46.3% were deformed in the ∆*Aomdr1* mutants and 13.2% were deformed in the WT strain (Figure 4D). The conidia yield was considerably reduced to 85,000 spores per mL for the ∆*Aomdr1* mutants and 136,000 spores per mL for the WT strain (Figure 4E).

### 3.6. AoMdr1 Regulates the Number of Traps and Trap Morphology

**Figure 5:**
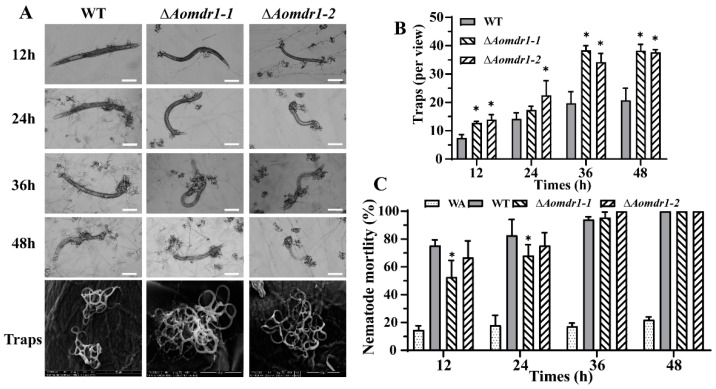
Comparisons of trap formation, morphology, and nematode predation efficiency. (**A**) Trap formation at different time points; scale bar: 50 μm. (**B**) Numbers of traps at different times of nematode induction. (**C**) Nematode mortality at different times. Asterisks indicate significant differences between the ∆*Aomdr1* mutant and WT strains (Tukey HSD; * *p* < 0.05).

At different times in nematode induction, both the WT and mutant strains were able to form traps to capture the nematodes. The traps produced by the ∆*Aomdr1* mutants contained greater numbers of mycelial loops (Figure 5A). The numbers of traps produced by the ∆*Aomdr1* mutants were higher than those produced by the WT strain at all time points. There were 7.5 and 13.4 traps per field of view at 12 h for the WT and ∆*Aomdr1* mutants, respectively, and 14.2 and 19.9 traps, respectively, per field of view at 24 h (Figure 5B), whereas there was no obvious difference in nematode mortality between the ∆*Aomdr1* mutant and WT strains at 12 or 24 h (Figure 5C). The extracellular proteolytic activity of the ∆*Aomdr1* mutants was lower than that of the WT strain (Figure S2C).

### 3.7. AoMdr1 Impairs Secondary Metabolites

**Figure 6:**
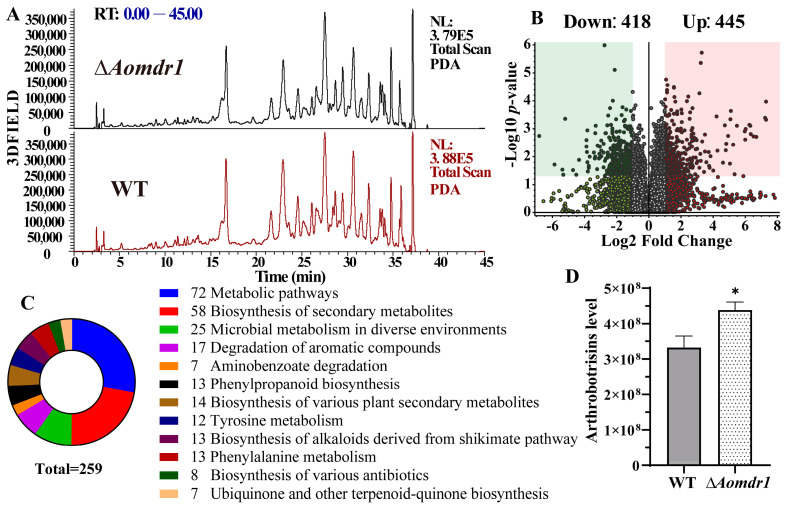
Comparisons of metabolic profiles of the WT and ∆*Aomdr1* mutant strains. (**A**) Comparison of high-performance liquid chromatography profiles of the WT and ∆*Aomdr1* mutants. (**B**) Analysis of the number of differentially up- and downregulated compounds. (**C**) KEGG enrichment analysis of differential compounds. (**D**) Comparison of the content of arthrobotrisins. Asterisks indicate significant differences between the ∆*Aomdr1* mutant and WT strains (Tukey HSD; * *p* < 0.0 5).

The compounds of the WT and mutant strains were extracted and analyzed using LC-MS. The metabolic profiles of the WT and mutant strains showed no obvious changes (Figure 6A), whereas the volcano plot of the metabolic data analysis revealed that 418 compounds were downregulated and 445 compounds were upregulated in the ∆*Aomdr1* mutants compared to in the WT (Figure 6B). A KEGG enrichment analysis of the differential compounds showed that 259 compounds were mainly enriched in various metabolic pathways (Figure 6C); these compounds were mainly distributed in the biosynthesis of secondary metabolites, alkaloids, and antibiotics as well as phenylalanine metabolism and ubiquinone and other terpenoid quinone. A quantitative analysis of arthrobotrisin was also performed; its content in the ∆*Aomdr1* mutants increased by 32% compared to in the WT (Figure 6D).

## 4. Discussion

Mdr proteins are critical for pathogenic fungi to be capable of drug resistance. They contain both ABC_tran and two ABC_membrane structural domains in different fungi [2] as well as high similarities in protein sequences, indicating that they are extremely conserved in different fungi. Previous studies have shown that Mdr1 is not only involved in drug resistance but also in regulating the pathogenicity of fungi; it has an important role in growth, development, and adaptation to stressful environments [45,46,47]. In this study, an orthologous Mdr protein, AoMdr1, was identified in the NT fungus *A. oligospora*, and its function was investigated. Our results show that AoMdr1 has important regulatory roles in mycelial growth, fusion, stress tolerance, LD accumulation, sporulation, and trap formation.

Cell-to-cell communication is essential for the formation of multiple connected filamentous fungi that build colony structures by fusion of hyphae or conidia [48]. Fusion is a highly dynamic and regulated process, and cellular communication regulates it [49]. The trap formation of *A. oligospora* initially involves annular structures, of which multiple are gradually formed and combine to form a three-dimensional adhesive network; the combination of these annular structures is closely linked to their mycelial fusion with each other [50]. Recently, we observed that the transcription factor Ste12 is required for mycelial fusion in *A. oligospora*, and that Fus3-MAPK and five other proteins (Mdr1, Mae1, Vps18, Ubx5, and UDP-glycosyltransferase) could interact with Ste12 [51]. In this study, the deletion of *Aomdr1* caused a reduction in mycelial fusion under nutrient-deficient conditions. Our results are similar to those for *Aomae1*; the deletion of *Aomdr1* also reduced mycelial fusion in nutrient-deficient media, whereas the fusion was unaffected in nutrient-rich media [33]. These similar results may indicate that AoMdr1 and AoMae1 are consistent in their regulation of mycelial fusion, both having a regulatory effect on mycelial fusion under low-nutrient conditions, suggesting that nutrients play a vital role in mycelial fusion under nutrient-deficient conditions.

In the present study, the disruption of the *Aomdr1* gene affected the mycelial growth and stress response of *A. oligospora*. The ∆*Aomdr1* mutants showed faster growth compared to the WT strains in the PDA, TG, and TYGA media. The ∆*Aomdr1* mutants also showed a degree of resistant growth under hyperosmotic stress and were more sensitive after the Flc and SDS treatments. It had previously been determined that Flc resistance in *C. albicans* occurs due to Mdr1 activation by the green fluorescent protein (GFP)-labeling of the Mdr transporter protein [52]. Overexpression of *mdr1* has induced high-level Flc resistance in *C. tropicalis* clinical isolates [5]. Upregulation of *mdr1* expression has also been particularly important in the azole resistance of *Candida auris* [53]. In *Cryptococcus gattii* and *C. neoformans*, the Mdr1 efflux pump was strongly associated with azole resistance [54]. These similar results illustrate the involvement of AoMdr1 in regulating the Flc resistance and stress response of *A. oligospora*.

Previous studies have suggested that shortened cell length and increased LD accumulation are closely related to trap formation [33]. In this study, the deletion of *Aomdr1* caused a considerable increase in mycelial septa and shortened the cell length in the mutant strain, which led to accelerated and increased trap formation in the ∆*Aomdr1* mutants. These results are consistent with those for AoMae1 and indicate that the disruption of *Aomdr1* caused a significant shortening in cell length, further affecting the formation of traps. LDs are central organelles for lipid and energy conversion, and their biogenesis and degradation are closely related to levels of cellular metabolism [55]. The nucleus is the control center for all cellular life activities. In this study, we also detected a reduction in LD accumulation and a decrease in the number of nuclei. These results suggest that AoMdr1 has critical functions in septum distribution, nuclei formation, and lipid metabolism, resulting in regulation of the mycelial development and trap formation of *A. oligospora*.

ABC transporters contribute, in different respects, to antioxidant capacity; several of them are necessary for the full virulence of *Beauveria bassiana* [47]. Importantly, the deletion of the *Aomdr1* gene in this study resulted in a significant increase in the number of traps, and the traps produced by the ∆*Aomdr1* mutants contained greater numbers of mycelial loops. The nematode predation efficiencies of the ∆*Aomdr1* mutants showed no obvious changes, suggesting that trapping nematodes requires the involvement of not only traps but also sticky substances on the surfaces of the traps, as well as extracellular proteases. Disruption of *Aoste12* has caused a significant increase in numbers of mycelial loops in traps, but has decreased numbers of traps [51]. The absence of AoMae1, another protein that interacts with AoSte12, has led to increases in the numbers of traps and the numbers of mycelial loops [33]. These results indicate that deletion of *Aoste12* and interacting genes triggers an increase in the number of mycelial loops contained in traps, suggesting that these factors regulate trapped mycelial loops in a similar way. The differences in the numbers of traps appear to indicate that the three genes have different roles in trap formation.

Conidiation is important for the survival and pathogenicity of NT fungi [37]. The conidial production of the ∆*Aomdr1* mutants decreased by 37.5%; 33.1% of the conidia were deformed in the ∆*Aomdr1* mutants, which is consistent with the variations in spore production and morphology caused by *Aoste12* deletion. This further suggests that *Aomdr1* may be a downstream target gene of *Aoste12*, and that *Aomdr1* is closely related to conidia formation. The metabolomic analysis showed that 863 compounds appeared to be differentially expressed in the ∆*Aomdr1* mutants, and the content of arthrobotrisins increased by 32%. Arthrobotrisins are a special group of metabolites identified from *A. oligospora* and other NT fungi and are involved in mycelial development and trap formation [56,57]. Thus, we concluded that AoMdr1 plays a crucial role in the conidiation and secondary metabolism of *A. oligospora*.

## 5. Conclusions

Our results show that AoMdr1 contributes to the fluconazole resistance of *A. oligospora* by regulating cell membrane transport, thus enabling it to respond to osmotic stress. It also impairs LD accumulation, cell septa, nuclei, and mycelial fusion, leading to changes in conidial production and morphology. It is also involved in the trap formation of *A. oligospora*. In summary, our study revealed, for the first time, the roles of AoMdr1 in mycelial development, fluconazole resistance, conidiation, and the trap formation of *A. oligospora*, providing insight into the role of Mdr proteins in filamentous fungi.

## Data Availability

The data presented in this study are available in supplementary material.

## Supplementary Materials

The following supporting information can be downloaded at: https://www.mdpi.com/article/10.3390/microorganisms11061612/s1, Figure S1. Phylogenetic analysis and validation of *Aomdr1* knockout strain. Figure S2. Comparison of plasma-wall separation, stress response to heat shock, and extracellular proteolytic activities of WT and mutant strains. Table S1. List of primers used in this study. Table S2. Information on the plasmids used in this study.

## References

[1] Nazarov P.A. (2022). MDR pumps as crossroads of resistance: Antibiotics and bacteriophages. Antibiotics.

[2] Cavalheiro M., Pais P., Galocha M., Teixeira M.C. (2018). Host-pathogen interactions mediated by MDR transporters in fungi: As pleiotropic as it gets!. Genes.

[3] Wegner M.S., Gruber L., Mattjus P., Geisslinger G., Grosch S. (2018). The UDP-glucose ceramide glycosyltransferase (UGCG) and the link to multidrug resistance protein 1 (MDR1). BMC Cancer.

[4] Liu Z.L., Myers L.C. (2017). *Candida albicans* Swi/Snf and mediator complexes differentially regulate Mrr1-induced MDR1 expression and fluconazole resistance. Antimicrob. Agents Chemother..

[5] Jin L.Y., Cao Z.R., Wang Q., Wang Y.C., Wang X.J., Chen H.B., Wang H. (2018). MDR1 overexpression combined with ERG11 mutations induce high-level fluconazole resistance in *Candida tropicalis* clinical isolates. BMC Infect. Dis..

[6] Du W.L., Zhai P.F., Wang T.L., Bromley M.J., Zhang Y.W., Lu L. (2021). The C_2_H_2_ transcription factor SltA contributes to azole resistance by coregulating the expression of the drug target Erg11A and the drug efflux pump Mdr1 in *Aspergillus fumigatus*. Antimicrob. Agents Chemother..

[7] Fattahi A., Zaini F., Kordbacheh P., Rezaie S., Safara M., Fateh R., Farahyar S., Kanani A., Heidari M. (2015). Evaluation of mRNA expression Levels of *cyp51A* and *mdr1*, candidate genes for voriconazole resistance in *Aspergillus flavus*. Jundishapur. J. Microbiol..

[8] Yang M.L., Uhrig J., Vu K., Singapuri A., Dennis M., Gelli A., Thompson G.R. (2016). Fluconazole susceptibility in *Cryptococcus gattii* is dependent on the ABC transporter Pdr11. Antimicrob. Agents Chemother..

[9] Jiang X.Z., Xiang M.C., Liu X.Z. (2017). Nematode-trapping fungi. Microbiol. Spectr..

[10] Su H., Zhao Y., Zhou J., Feng H.H., Jiang D.W., Zhang K.Q., Yang J.K. (2017). Trapping devices of nematode-trapping fungi: Formation, evolution, and genomic perspectives. Biol. Rev. Camb. Philos. Soc..

[11] Zhu M.C., Li X.M., Zhao N., Yang L., Zhang K.Q., Yang J.K. (2022). Regulatory mechanism of trap formation in the nematode-trapping fungi. J. Fungi.

[12] Yang E.C., Xu L.L., Yang Y., Zhang X.Y., Xiang M.C., Wang C.S., An Z.Q., Liu X.Z. (2012). Origin and evolution of carnivorism in the *Ascomycota* (fungi). Proc. Natl. Acad. Sci. USA.

[13] Wang X., Li G.H., Zou C.G., Ji X.L., Liu T., Zhao P.J., Liang L.M., Xu J.P., An Z.Q., Zheng X. (2014). Bacteria can mobilize nematode-trapping fungi to kill nematodes. Nat. Commun..

[14] Yang J.K., Wang L., Ji X.L., Feng Y., Li X.M., Zou C.G., Xu J.P., Ren Y., Mi Q.L., Wu J.L. (2011). Genomic and proteomic analyses of the fungus *Arthrobotrys oligospora* provide insights into nematode-trap formation. PLoS Pathog..

[15] Zhou D.X., Zhu Y.M., Bai N., Yang L., Xie M.H., Yang J.L., Zhu M.C., Zhang K.Q., Yang J.K. (2022). AoATG5 plays pleiotropic roles in vegetative growth, cell nucleus development, conidiation, and virulence in the nematode-trapping fungus *Arthrobotrys oligospora*. Sci. China Life Sci..

[16] Chen Y.L., Gao Y., Zhang K.Q., Zou C.G. (2013). Autophagy is required for trap formation in the nematode-trapping fungus *Arthrobotrys oligospora*. Environ. Microbiol. Rep..

[17] Li X.M., Zhu M.C., Liu Y.K., Yang L., Yang J.K. (2023). *Aoatg11* and *Aoatg33* are indispensable for mitophagy, and contribute to conidiation, the stress response, and pathogenicity in the nematode-trapping fungus *Arthrobotrys oligospora*. Microbiol. Res..

[18] Zhou D.X., Zhu Y.M., Bai N., Xie M.H., Zhang K.Q., Yang J.K. (2022). *Aolatg1* and *Aolatg13* regulate autophagy and play different roles in conidiation, trap formation, and pathogenicity in the nematode-trapping fungus *Arthrobotrys oligospora*. Front. Cell. Infect. Microbiol..

[19] Liu Q.Q., Li D.N., Bai N., Zhu Y.M., Yang J.K. (2023). Peroxin Pex14/17 is required for trap formation, and plays pleiotropic roles in mycelial development, stress response, and secondary metabolism in *Arthrobotrys oligospora*. mSphere.

[20] Liu Q.Q., Li D.N., Jiang K.X., Zhang K.Q., Yang J.K. (2022). AoPEX1 and AoPEX6 are required for mycelial growth, conidiation, stress response, fatty acid utilization, and trap formation in *Arthrobotrys oligospora*. Microbiol. Spectr..

[21] Bai N., Zhang G.S., Wang W.J., Feng H.H., Yang X.W., Zheng Y.Q., Yang L., Xie M.H., Zhang K.Q., Yang J.K. (2022). Ric8 acts as a regulator of G-protein signalling required for nematode-trapping lifecycle of *Arthrobotrys oligospora*. Environ. Microbiol..

[22] Ma N., Zhao Y.N., Wang Y.C., Yang L., Li D.N., Yang J.L., Jiang K.X., Zhang K.Q., Yang J.K. (2021). Functional analysis of seven regulators of G protein signaling (RGSs) in the nematode-trapping fungus *Arthrobotrys oligospora*. Virulence.

[23] Wang W.J., Zhao Y.N., Bai N., Zhang K.Q., Yang J.K. (2022). AMPK is involved in regulating the utilization of carbon sources, conidiation, pathogenicity, and stress response of the nematode-trapping fungus *Arthrobotrys oligospora*. Microbiol. Spectr..

[24] Chen S.A., Lin H.C., Schroeder F.C., Hsueh Y.P. (2021). Prey sensing and response in a nematode-trapping fungus is governed by the MAPK pheromone response pathway. Genetics.

[25] Xie M.H., Yang J.L., Jiang K.X., Bai N., Zhu M.C., Zhu Y.M., Zhang K.Q., Yang J.K. (2021). AoBck1 and AoMkk1 are necessary to maintain cell wall integrity, vegetative growth, conidiation, stress resistance, and pathogenicity in the nematode-trapping fungus *Arthrobotrys oligospora*. Front. Microbiol..

[26] Zhen Z.Y., Xing X.J., Xie M.H., Yang L., Yang X.W., Zheng Y.Q., Chen Y.L., Ma N., Li Q., Zhang K.Q. (2018). MAP kinase Slt2 orthologs play similar roles in conidiation, trap formation, and pathogenicity in two nematode-trapping fungi. Fungal Genet. Biol..

[27] Christianson T.W., Sikorski R.S., Dante M., Shero J.H., Hieter P. (1992). Multifunctional yeast high-copy-number shuttle vectors. Gene.

[28] Jiang D.W., Zhou J., Bai G.Z., Xing X.J., Tang L.Y., Yang X.W., Li J., Zhang K.Q., Yang J.K. (2017). Random mutagenesis analysis and identification of a novel C(_2_)H(_2_)type transcription factor from the nematode-trapping fungus *Arthrobotrys oligospora*. Sci. Rep..

[29] Ma Y.X., Yang X.W., Xie M.H., Zhang G.S., Yang L., Bai N., Zhao Y.N., Li D.N., Zhang K.Q., Yang J.K. (2020). The Arf-GAP AoGlo3 regulates conidiation, endocytosis, and pathogenicity in the nematode-trapping fungus *Arthrobotrys oligospora*. Fungal Genet. Biol..

[30] Kumar S., Stecher G., Tamura K. (2016). MEGA7: Molecular evolutionary genetics analysis version 7.0 for bigger datasets. Mol. Biol. Evol..

[31] Colot H.V., Park G., Turner G.E., Ringelberg C., Crew C.M., Litvinkova L., Weiss R.L., Borkovich K.A., Dunlap J.C. (2006). A high-throughput gene knockout procedure for *Neurospora* reveals functions for multiple transcription factors. Proc. Natl. Acad. Sci. USA.

[32] Fan Y., Zhang W.W., Chen Y., Xiang M.C., Liu X.Z. (2021). DdaSTE12 is involved in trap formation, ring inflation, conidiation, and vegetative growth in the nematode-trapping fungus *Drechslerella dactyloides*. Appl. Microbiol. Biotechnol..

[33] Liu Y., Zhu M., Wang W., Li X., Bai N., Xie M., Yang J. (2023). AoMae1 regulates hyphal fusion, lipid droplet accumulation, conidiation, and trap formation in *Arthrobotrys oligospora*. J. Fungi.

[34] Yang X.W., Ma N., Yang L., Zheng Y.Q., Zhen Z.Y., Li Q., Xie M.H., Li J., Zhang K.Q., Yang J.K. (2018). Two Rab GTPases play different roles in conidiation, trap formation, stress resistance, and virulence in the nematode-trapping fungus *Arthrobotrys oligospora*. Appl. Microbiol. Biotechnol..

[35] Jiang K.X., Liu Q.Q., Bai N., Zhu M.C., Zhang K.Q., Yang J.K. (2022). AoSsk1, a response regulator required for mycelial growth and development, stress responses, trap formation, and the secondary metabolism in *Arthrobotrys oligospora*. J. Fungi.

[36] Zhu Y.M., Zhou D.X., Bai N., Liu Q.Q., Zhao N., Yang J.K. (2023). SNARE protein AoSec22 orchestrates mycelial growth, vacuole assembly, trap formation, stress response, and secondary metabolism in *Arthrobotrys oligospora*. J. Fungi.

[37] Zhang G.S., Zheng Y.Q., Ma Y.X., Yang L., Xie M.H., Zhou D.X., Niu X.M., Zhang K.Q., Yang J.K. (2019). The velvet proteins VosA and VelB play different roles in conidiation, trap formation, and pathogenicity in the nematode-trapping fungus *Arthrobotrys oligospora*. Front Microbiol..

[38] Liu J., Tong S.M., Qiu L., Ying S.H., Feng M.G. (2017). Two histidine kinases can sense different stress cues for activation of the MAPK Hog1 in a fungal insect pathogen. Environ. Microbiol..

[39] Liu J., Wang Z.K., Sun H.H., Ying S.H., Feng M.G. (2017). Characterization of the Hog1 MAPK pathway in the entomopathogenic fungus *Beauveria bassiana*. Environ. Microbiol..

[40] Yang J.L., Wang W.J., Liu Y.K., Xie M.H., Yang J.K. (2023). The MADS-box transcription factor AoRlmA is involved in the regulation of mycelium development, conidiation, cell-wall integrity, stress response, and trap formation of *Arthrobotrys oligospora*. Microbiol. Res..

[41] Yang L., Li X.M., Xie M.H., Bai N., Yang J.L., Jiang K.X., Zhang K.Q., Yang J.K. (2021). Pleiotropic roles of Ras GTPases in the nematode-trapping fungus *Arthrobotrys oligospora* identified through multi-omics analyses. Iscience.

[42] Xie M.H., Ma N., Bai N., Yang L., Yang X.W., Zhang K.Q., Yang J.K. (2022). PKC-SWI6 signaling regulates asexual development, cell wall integrity, stress response, and lifestyle transition in the nematode-trapping fungus *Arthrobotrys oligospora*. Sci. China Life Sci..

[43] Zhou J., Wu Q.F., Li S.H., Yan J.X., Wu L., Cheng Q.Y., He Z.Q., Yue X.T., Zhang K.Q., Zhang L.L. (2022). The multifaceted Gene 275 embedded in the PKS-PTS gene cluster was involved in the regulation of arthrobotrisin biosynthesis, TCA cycle, and septa formation in nematode-trapping fungus *Arthrobotrys oligospora*. J. Fungi.

[44] Xie M.H., Ma N., Bai N., Zhu M.C., Zhang K.Q., Yang J.K. (2022). Phospholipase C (AoPLC2) regulates mycelial development, trap morphogenesis, and pathogenicity of the nematode-trapping fungus *Arthrobotrys oligospora*. J Appl. Microbiol..

[45] Schubert S., Barker K.S., Znaidi S., Schneider S., Dierolf F., Dunkel N., Aid M., Boucher G., Rogers P.D., Raymond M. (2011). Regulation of efflux pump expression and drug resistance by the transcription factors Mrr1, Upc2, and Cap1 in *Candida albicans*. Antimicrob. Agents Chemother..

[46] Zhang Y., He K., Guo X.H., Jiang J., Qian L., Xu J.Q., Che Z.P., Huang X.B., Liu S.M. (2023). Transcriptomic profiling of *Fusarium pseudograminearum* in response to carbendazim, pyraclostrobin, tebuconazole, and phenamacril. J. Fungi.

[47] Song T.T., Zhao J., Ying S.H., Feng M.G. (2013). Differential contributions of five ABC Transporters to mutidrug resistance, antioxidion and virulence of *Beauveria bassiana*, an entomopathogenic fungus. PLoS ONE.

[48] Jonkers W., Fischer M.S., Do H.P., Starr T.L., Glass N.L. (2016). Chemotropism and cell fusion in *Neurospora crassa* Relies on the formation of distinct protein complexes by HAM-5 and a novel protein HAM-14. Genetics.

[49] Herzog S., Schumann M.R., Fleissner A. (2015). Cell fusion in *Neurospora crassa*. Curr. Opin. Microbiol..

[50] Nordbringhertz B., Friman E., Veenhuis M. (1989). Hyphal fusion during initial stages of trap formation in *Arthrobotrys oligospora*. Antonie Van Leeuwenhoek.

[51] Bai N., Xie M., Liu Q., Wang W., Liu Y., Yang J. (2023). AoSte12 is required for mycelial development, conidiation, trap morphogenesis, and secondary metabolism by regulating hyphal fusion in nematode-trapping fungus *Arthrobotrys oligospora*. Microbiol. Spectr..

[52] Suchodolski J., Krasowska A. (2021). Fructose induces fluconazole resistance in *Candida albicans* through activation of Mdr1 and Cdr1 transporters. Int. J. Mol. Sci..

[53] Li J.Z., Coste A.T., Bachmann D., Sanglard D., Lamoth F. (2022). Deciphering the Mrr1/Mdr1 pathway in azole resistance of *Candida auris*. Antimicrob. Agents Chemother..

[54] Basso L.R., Gast C.E., Bruzual I., Wong B. (2015). Identification and properties of plasma membrane azole efflux pumps from the pathogenic fungi *Cryptococcus gattii* and *Cryptococcus neoformans*. J. Antimicrob. Chemother..

[55] Olzmann J.A., Carvalho P. (2019). Dynamics and functions of lipid droplets. Nat. Rev. Mol. Cell Biol..

[56] Wei L.X., Zhang H.X., Tan J.L., Chu Y.S., Li N., Xue H.X., Wang Y.L., Niu X.M., Zhang Y., Zhang K.Q. (2011). Arthrobotrisins A-C, oligosporons from the nematode-trapping fungus *Arthrobotrys oligospora*. J. Nat. Prod..

[57] Yu X., Hu X., Pop M., Wernet N., Kirschhöfer F., Brenner-Weiß G., Keller J., Bunzel M., Fischer R. (2021). Fatal attraction of *Caenorhabditis elegans* to predatory fungi through 6-methyl-salicylic acid. Nat. Commun..

