# Supplementary material for: Involvement of AoMdr1 in the Regulation of the Fluconazole Resistance, Mycelial Fusion, Conidiation, and Trap Formation of Arthrobotrys oligospora
Source: Microorganisms. 2023 Jun 19;11(6):1612. doi: 10.3390/microorganisms11061612 (PMC10302927; doi:10.3390/microorganisms11061612)
Supplement: Supplementary file 1 [file microorganisms-11-01612-s001.zip › microorganisms-2440654-supplementary.pdf]

Supporting Information

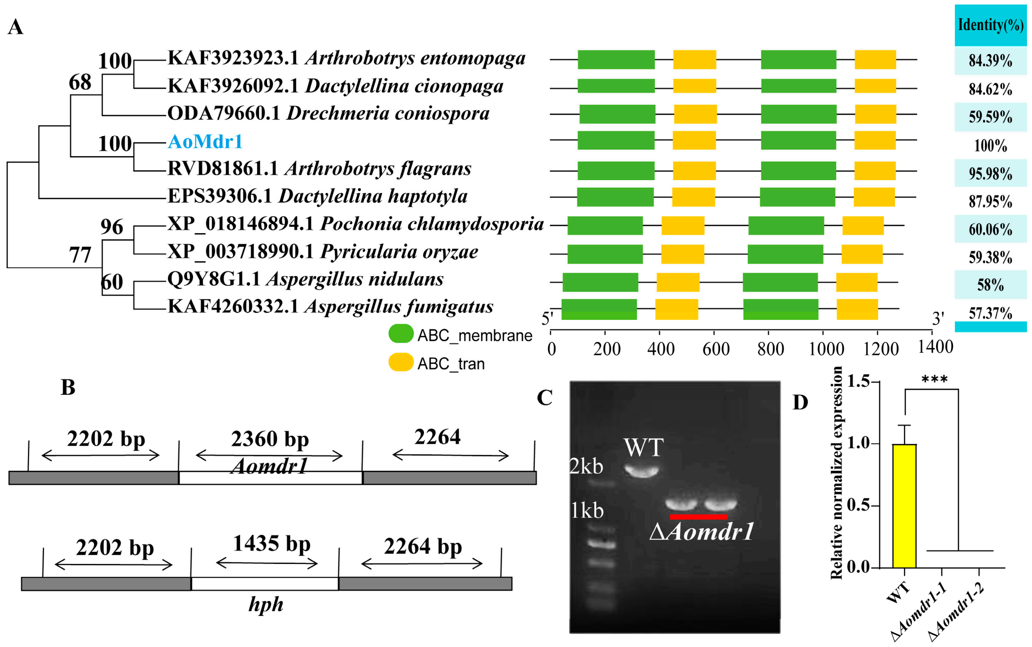

Figure S1. Phylogenetic analysis and validation of *Aomdr1* knockout strain. (A) Phylogenetic and structure domain analyses of Mdr1 orthologs from different fungi. (B) Diagram of the principle of homologous recombination. (C-D) Validation of knockout strains by PCR and RT-qPCR. In PCR analysis, the targeted bands of the *Aomdr1* knockout strains were marked with red lines. Asterisks indicate significant differences between the Δ*Aomdr1* mutant and WT strains (Tukey's HSD, ***$p < 0.01$).

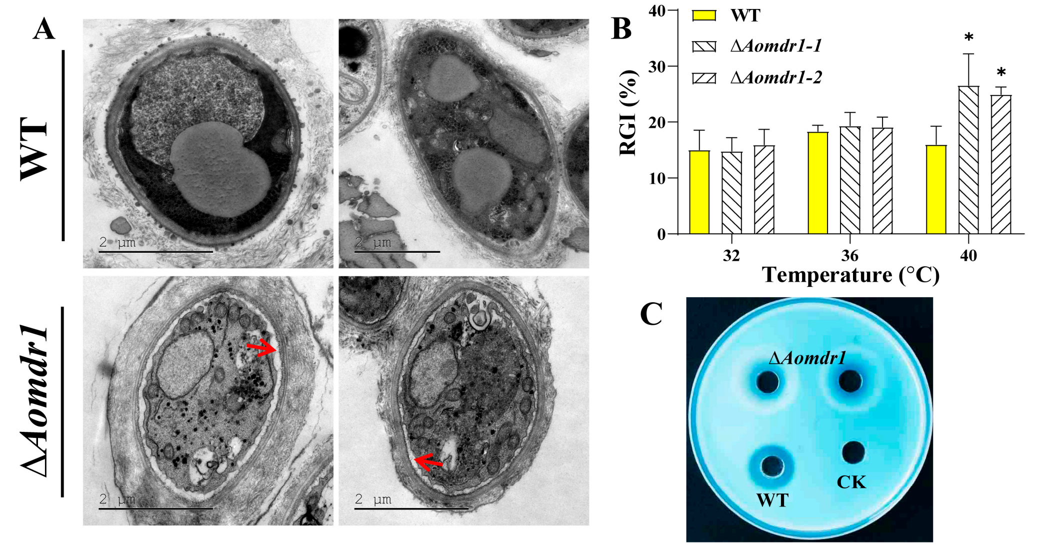

Figure S2. Comparison of plasma-wall separation, stress response to heat shock, and extracellular proteolytic activities of WT and mutant strains. (A) Plasma-wall separation observed by TEM. (B) Heat sensitivity of mutant strains. Asterisks indicate significant differences between the Δ*Aomdr1* mutant and WT strains (Tukey HSD, *$p < 0.05$). (C) Comparison of the extracellular protease activities of WT and mutant strains. CK, the PD broth was used as a control sample.

Table S1. List of primers used for gene manipulation in this study.

Primers	Primer sequence	Application
AoMdr1-5F	F-GTAACGCCAGGGTTTTCCCAGTCACGACGTACTGGAGTTACGTGATGCTGG	5' Homologous arm
AoMdr1-5R	R-ATCCACTTAACGTTACTGAAATCTCCAACTCGTACCCAAGCTTTCAGGA	
AoMdr1-3F	F-CTCCTTCAATATCATCTTCTGTCTCCGACTGGCGGTCTGTCTCCTGATA	3' Homologous arm
AoMdr1-3R	R-GCGGATAACAATTTCACACAGGAAACAGCTGTGAATGATGTCTGTCGAGT	
AoMdr1-PF	F-TCCTGAAAGCTTGGGTACGA	Transformant vernfication
AoMdr1-PR	R-TATCAGGAGACAGACCGCCA	
AoMdr1-RF	F-ATGATATACATTGCAGACCA	RT-PCR vernfication
AoMdr1-RR	R-CGTTCAATACAGCCTTTGGA	
Hph-F	F-GTTGGAGATTTCAGTAACGTTAAGTGGAT	
Hph-R	R-GTCGGAGACAGAAGATGATATTGAAGGAGC	Amplify the *hph*

Table S2. Information of the plasmids used in this study.

Plasmids	Selection marker	Application
pSCN44	Hph^+	For hygromycin resistance
pRS426	Amp^+	For constructing the disruption fragment
